# Same Same but Different. Different Trigeminal Chemoreceptors Share the Same Central Pathway

**DOI:** 10.1371/journal.pone.0121091

**Published:** 2015-03-16

**Authors:** Kathrin Kollndorfer, Ksenia Kowalczyk, Johannes Frasnelli, Elisabeth Hoche, Ewald Unger, Christian A. Mueller, Jacqueline Krajnik, Siegfried Trattnig, Veronika Schöpf

**Affiliations:** 1 Department of Biomedical Imaging and Image-guided Therapy, Medical University of Vienna, Vienna, Austria; 2 Department of Pediatric and Adolescent Medicine, Medical University of Vienna, Vienna, Austria; 3 Centre de Recherche en Neuropsychologie et Cognition, Département de Psychologie, Université de Montréal, Montréal, Canada; 4 Centre de Recherche, Hôpital du Sacre Coeur de Montréal, Montréal, Canada; 5 Center for Medical Physics and Biomedical Engineering, Medical University of Vienna, Vienna, Austria; 6 Department of Otorhinolaryngology, Medical University of Vienna, Vienna, Austria; 7 High Field MR Center, Department of Biomedical Imaging and Image-guided Therapy, Medical University of Vienna, Vienna, Austria; University of South California, UNITED STATES

## Abstract

Intranasal trigeminal sensations are important in everyday life of human beings, as they play a governing role in protecting the airways from harm. Trigeminal sensations arise from the binding of a ligand to various sub-types of transient receptor potential (TRP) channels located on mucosal branches of the trigeminal nerve. Which underlying neural networks are involved in the processing of various trigeminal inputs is still unknown. To target this unresolved question fourteen healthy human subjects were investigated by completing three functional magnetic resonance imaging (fMRI) scanning sessions during which three trigeminal substances, activating varying sub-types of chemoreceptors and evoking different sensations in the nose were presented: CO_2_, menthol and cinnamaldehyde. We identified similar functional networks responding to all stimuli: an olfactory network, a somatosensory network and an integrative network. The processing pathway of all three stimulants was represented by the same functional networks, although CO_2_ evokes painful but virtually odorless sensations, and the two other stimulants, menthol and cinnamaldehyde are perceived as mostly non painful with a clear olfactory percept. Therefore, our results suggest a common central processing pathway for trigeminal information regardless of the trigeminal chemoreceptor and sensation type.

## Introduction

Chemosensory perception is, in part, the result of the interaction between the olfactory and the trigeminal system. Whereas the olfactory system is responsible for the perception of the quality of odors, the latter conveys sensations such as burning, stinging, pungency, temperature, or pain and thereby serves as an additional sentinel to protect the airways from harm [1–5].

Intranasal trigeminal stimulation evokes neuronal activation of pain processing areas, including the anterior cingulate cortex, the insula, or the primary somatosensory cortex [6–8], as well as chemosensory processing regions, such as the orbitofrontal cortex [9]. It has recently been shown that trigeminal perception arises from the interaction of a ligand with specific receptors, similar to the olfactory system. So far, various sub-families of transient receptor potential (TRP) channels have been identified to transmit chemosensory information as well as temperature changes. For example, the TRPV1 receptor responds to heat and to compounds, such as capsaicin or carbon dioxide (CO_2_); its activation leads to a burning and stinging sensation [10,11]. Another example is the TRPM8 receptor, activated by cool temperatures and compounds such as menthol or eucalyptol; creating a cooling sensation [12,13]. A third example is the TRPA1 receptor, which is stimulated by both cold temperatures and substances such as mustard oil or cinnamaldehyde [14,15]; its activation evokes a burning cold sensation. Interestingly, TRPV1 and TRPA1 receptors are often expressed in the same sensory neurons [16,17], whereas the TRPA1 and the TRPM8 are exclusively expressed in different sensory neurons [17]. This has direct functional consequences, as sensitivity to agonists of different trigeminal receptors is not distributed equally in the population [18]. While past studies have provided an increased comprehension of the periphery of the trigeminal system, we do not yet understand the mechanisms that occur during the central processing of trigeminal perception. We, therefore, aimed to investigate central activation patterns evoked by agonists of three different trigeminal chemoreceptors; specifically, we compared: 1) CO_2_ (activating TRPV1); 2) menthol (stimulating TRPM8); and 3) cinnamaldehyde (activating TRPA1). We hypothesized that we would observe largely overlapping activation patterns with receptor-specific differences.

Functional imaging data was analyzed conducting group independent component analysis (ICA) for all three scanning sessions to compare the determined processing networks for trigeminal stimulation, as previous studies investigating fMRI analyzing techniques in the context of chemosensory experiments revealed the high susceptibility to movement artifacts [19,20] in hypothesis-driven methods like the General Linear Model (GLM).

## Materials and Methods

### Stimulus rating

To ensure not only the binding of the substances to various chemoreceptors but also the different sensations evoked by the investigated stimuli, all three odorants were evaluated separately. All stimuli were rated by a subject group of 9 raters (7 female, 2 male; mean age, 27.4 years, SD, 3.3), who did not participate in the fMRI experiment. The evaluation was assessed on a visual analogue scale (VAS) ranging from 0 to 100. The stimulus rating included the assessment of pleasantness, painfulness and familiarity of the applied substance. Furthermore, the raters were asked to label the stimulus.

### Subjects

Nineteen healthy subjects (ten female, nine male) participated in our study. All subjects had normal olfactory function (for details see olfactory performance section) and had no history of neurological or psychiatric diseases, which were known to influence olfactory performance. All subjects did not report any severe head trauma in the past. Five subjects had to be excluded from the data set, as they did not complete all fMRI measurements. Fourteen subjects (seven female, seven male; mean age, 30.1 years; SD, 6.7) completed all measurements. All subjects were acquired by announcements at the Medical University of Vienna. The study was approved by the Ethics Committee of the Medical University of Vienna. All subjects were informed about the aim of the study and gave their written informed consent prior to inclusion.

### Olfactory performance

Psychophysiological measurement of olfactory performance was assessed using the Sniffin’ Sticks test battery (Burghart Instruments, Wedel, Germany). This test battery includes three subtests that assess nasal chemosensory function—detection threshold, odor discrimination and odor identification—using pen-like devices for odor presentation [21–23]. The olfactory detection threshold of n-butanol was assessed using a single-staircase, three-alternative, forced-choice procedure. Next, we determined odor discrimination ability using 16 triplets of odorants (two pens contained the same odorant; the third pen contained an odd odorant). The participants’ task was to detect the odd pen (forced choice). The odor identification task is composed of 16 common odors using a multiple-choice answering format with a list of four descriptors for each odor. The scores for the detection threshold can range from 1–16, for the other two subtests a score between 0 and 16 can be achieved. The results of all three subtests were summed to obtain the “TDI-score.” Normosmia, or normal olfactory performance, is characterized by a TDI-score of at least 31 [23].

### Chemosensory stimulation

All subjects of the fMRI experiment completed three scanning sessions, one for every stimulus: 1) CO_2_ (50% v/v); 2) cinnamaldehyde (75% v/v dissolved in 1,2-propanediol; Sigma-Aldrich, Germany); and 3) menthol (2.5g; Sigma-Aldrich, Germany). Trigeminal stimuli were delivered in an event-related design, using a computer-controlled, air-dilution olfactometer compatible with magnetic resonance imaging (MRI), which was constructed at the Center for Medical Physics and Biomedical Engineering (Medical University of Vienna). The mobile olfactometer with a dimension of 37x30x11cm and a total weight of 2.5kg comprises four separated airways channels for stimulation and provides two options of administering chemosensory stimuli; either by using custom-made odor reservoirs or gaseous stimuli, which can be directly connected to the device. These four airways are compounded of 2mm teflon tubes with an inner diameter of 1.5mm. By actuating a valve, air is diverted through and enters the odor reservoir. Each stimulation air-line is controlled by its own electromagnetic valve, which is realized with a 24V linear solenoid (type: 195227–231, Co. Saia-Burges) and a teflon membrane valve. To control the activation of the normally closed solenoid valve, a free programmable microcontroller (PIC16F690, Co. Microchip) in combination with a power output stage is used to define the stimulation sequence. The stimulation session is adjusted by three parameters: the channel number, the on time (stimulation) and the off time (no stimulation). After programming the microcontroller the olfactometer works with this setup without any additional support by a computer and can be started with a single start button. A second possibility for the isolated activation of the gas line is realized by a simple switch parallel connected to the output stage. This feature is used to test the function of the valves and to adjust the flow-rate (up to 1.5l/min) of the stimulation gas with the needle valve of the support gas. The flavoured gas output of all stimulation channels are add to a single air-line, which ends with a nose applicator.

### Imaging methods

FMRI measurements were performed on a 3 Tesla Trio System (Siemens Medical Solution, Erlangen, Germany) using a 32-channel head coil, and single-shot, gradient-recalled, echo-planar imaging (EPI). Thirty-six slices (2.7mm thickness, 0.5mm gap) were acquired, with a Field of View (FOV) of 210 x 210mm and an echo time (TE)/ repetition time (TR) of 32/2000ms. Slices were aligned parallel to the connection between the anterior and the posterior commissure. To correct for distortion, caused by inhomogeneity of the magnetic field, online distortion correction was performed with point-spread function mapping [24].

Participants were positioned in head first supine position in the scanner. The mobile olfactometer was placed outside the scanner room. A tube connected to the stimulation device was positioned into the vestibulum nasi without discomfort for the subject, in order to prevent nasal obstruction and was then attached to the head coil to avoid shifting of the tube during the measurements.

All subjects who participated in this study completed three scanning sessions with different trigeminal stimuli, each lasting ten minutes. All stimuli were applied monorhinally to the left nostril. Stimuli were presented for 500ms with an interstimulus interval (ISI) of 30s, resulting in 20 trigeminal pulses per scanning session. Stimuli were presented in pseudorandomized order to minimize interaction effects between substances. During fMRI measurements, subjects were asked to use a velopharyngeal closure breathing technique [25] and to keep their eyes closed [26] during all three scanning sessions.

### Data analysis

FMRI data were preprocessed using SPM8 (http://www.fil.ion.ucl.ac.uk/spm/), implemented in MATLAB (Matlab 7.14.0, Release 2012a, Mathworks Inc., Sherborn, MA, USA). Standard preprocessing in SPM contained 1) slice-timing, to correct for the temporal difference between the first and the last slice of the total volume; 2) motion correction to adjust for head movement; 3) spatial normalization, a registration of the individual brain to a standardized template of the Montreal Neurological Institute (MNI) to correct for individual brain shape and size; and 4) spatial smoothing, whereby small blurring kernels were applied to the image, in order to average parts of neighboring voxels and thereby softening hard edges.

ICA is a data-driven analysis method, which is used to separate a multivariate signal into independent components. Previous research has shown that ICA is especially suitable to analyze chemosensory experiments [19,20], as chemosensory paradigms are generally performed using an event-related design.

Group ICA was performed for all three scanning sessions conjointly using the Group ICA for fMRI Toolbox (GIFT; http://icatb.sourceforge.net/ [27]). The number of independent components (ICs) was estimated using the minimum description length (MDL) criterion [28], as implemented in GIFT. After dimension reduction using principal component analysis (PCA) in two reduction steps, group ICA was performed using the Infomax algorithm [29]. The statistical reliability of estimated ICs was tested using the ICASSO toolbox [30], implemented in GIFT. Using ICASSO, the IC estimation was calculated 20 times, varying the initial conditions of the algorithm as well as the bootstrapped datasets. The reliability of the identified ICs was assessed by clustering the results of each run. As a last step, we calculated differences between the three substances using a one-way ANOVA (FWE-corrected, p < 0.05) using SPM8.

For data visualization, whole-brain spatial activation maps were imported to the Multi-image analysis GUI (MANGO, http://ric.uthscsa.edu/mango) and overlaid onto a standardized anatomical template in MNI space.

## Results

### Olfactory performance

Olfactory testing revealed normal olfactory performance for all investigated participants. The achieved TDI-score ranged from 34 to 40.25 (mean TDI 36.00; SD, 2.05), and no significant differences were found between males (mean TDI 35.93; SD 2.56) and females (mean TDI 36.07; SD 1.59).

### Rating of trigeminal stimuli

The investigated stimuli were evaluated with regard to pleasantness, pain perception, familiarity on a visual analogue scale (VAS). The results of the ratings of the applied substances are presented in Table 1.

**Table 1:** Ratings of the three applied substances CO_2_, menthol and cinnamaldehyde.

	CO_2_ mean (SD)	menthol mean (SD)	Cinnamaldehyde mean (SD)	p-value^a^
pleasantness^b^	27.44 (23.42)	75.44 (18.98)	80.44 (17.57)	<. 001
pain^c^	59.56 (34.34)	4.00 (10.89)	1.22 (1.56)	<. 001
familiarity^b^	47.22 (32.34)	69.67 (24.50)	70.00 (31.73)	.240
label (%)^d^	0	66.67	66.67	

^a^ Bonferroni corrected

^b^ High values indicate pleasant/ familiar stimuli

^c^ High values indicate painful stimuli

^d^ Percentage of subjects who were able to label the substance correctly

### fMRI results

All three functional data sets, one for each session and stimulation, were submitted to a combined group ICA estimation, which resulted in 50 independent components. For further analysis, we included within-brain activations of the reported components.

For each stimulus, we observed three major networks, which overlapped largely between stimuli. These networks were labeled the (1) olfactory network, (2) somatosensory, and (3) integrative network.

**Fig 1:**
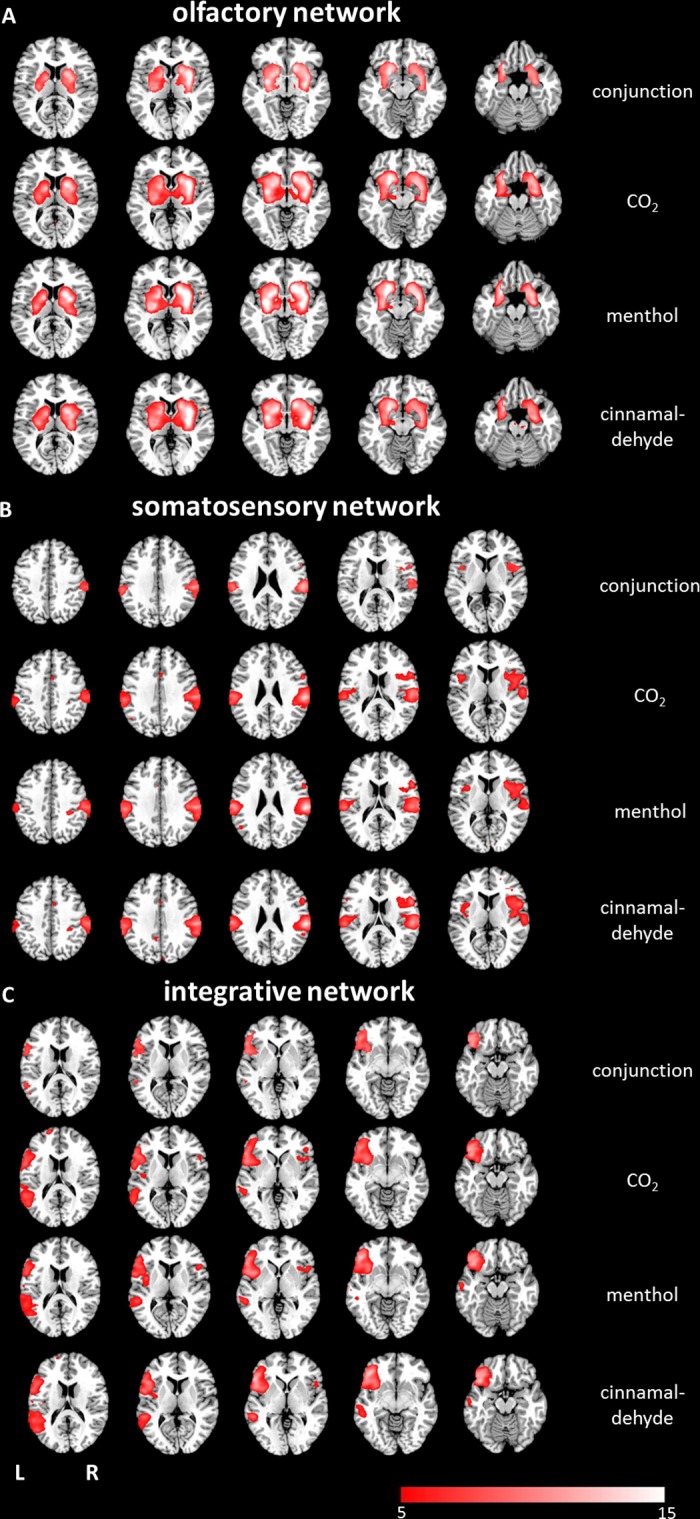
Axial mean anatomical images overlaid with (A) the olfactory network, (B) the somatosensory network and (C) the integrative network resulting from the combined group ICA (p < 0.05, FWE-corrected). All three networks were detected for all three investigated substances. For all investigated networks, the conjunction analysis revealed highly overlapping functional network patterns.

1) Olfactory network: This network was centered in the putamen, bilaterally, which comprised characteristic olfactory processing areas, including the piriform cortex (PIR), the entorhinal cortex, the amygdala, as well as parts of the thalamus (see Table 2). Importantly, this network was relatively symmetric in both hemispheres, even though the stimuli were only applied to the left nostril. We compared the spatial extent of this network for the three stimuli by using a one-way ANOVA, which revealed no significant differences (see Fig. 1A).

**Table 2:** Significant FWE- corrected clusters of the olfactory network for all substances separately and for the conjunction of three stimuli.

	Cluster size^a^	p- value^b^	x^c^	y^c^	z^c^	Anatomical label^d^
conjunction	4883	< 0.05	32	6	2	putamen
	4071	< 0.05	-30	-8	-6	putamen
CO_2_	10635	< 0.05	30	8	2	putamen
menthol	5362	< 0.05	30	-10	-2	putamen
	4545	< 0.05	-30	-8	-6	putamen
cinnamaldehyde	5885	< 0.05	30	-10	-2	putamen
	4609	< 0.05	-30	-8	-6	putamen

^a^ Significantly activated clusters with 10 or more voxels

^b^ p < 0.05 FWE-corrected at the cluster level

^c^ Coordinates in MNI space

^d^ Clusters were automatically labeled using the AAL toolbox [31].

2) Somatosensory network: The network comprised some areas of the pain matrix [32], such as the primary and secondary somatosensory cortex and the insula (see Table 3). Although neuronal activation was found bilaterally, clusters were significantly larger in the right hemisphere. Again, a one-way ANOVA revealed no significant differences between the spatial distributions of brain activation due to the three stimuli (see Fig. 1B).

**Table 3:** Significant FWE- corrected clusters of the somatosensory network for all substances separately and for the conjunction of three stimuli.

	Cluster size^a^	p- value^b^	x^c^	y^c^	z^c^	Anatomical label^c^
conjunction	1897	< 0.05	62	-28	26	supramarginal gyrus
	775	< 0.05	-58	-28	20	supramarginal gyrus
	629	< 0.05	56	12	16	inferior frontal gyrus (opercular part)
	64	< 0.05	-42	6	4	insula
CO_2_	2192	< 0.05	62	-28	26	supramarginal gyrus
	941	< 0.05	-58	-28	20	supramarginal gyrus
	860	< 0.05	48	0	6	rolandic operculum
	174	< 0.05	-42	10	4	insula
	31	< 0.05	14	-56	62	superior parietal lobule
	24	< 0.05	4	8	48	supplementary motor area
menthol	2369	< 0.05	64	-26	24	supramarginal gyrus
	1121	< 0.05	-58	-28	20	supramarginal gyrus
	1010	< 0.05	58	12	16	inferior frontal gyrus (opercular part)
	136	< 0.05	-42	8	2	insula
	104	< 0.05	30	-44	64	postcentral gyrus
	54	< 0.05	32	-36	42	postcentral gyrus
	13	< 0.05	42	6	-14	insula
cinnamaldehyde	3473	< 0.05	62	-28	26	supramarginal gyrus
	1063	< 0.05	-66	-24	26	supramarginal gyrus
	87	< 0.05	-42	6	4	insula
	25	< 0.05	6	8	48	supplementary motor area
	22	< 0.05	-40	-10	12	rolandic operculum
	20	< 0.05	32	-36	42	postcentral gyrus
	11	< 0.05	30	-44	62	postcentral gyrus
	10	< 0.05	38	34	6	inferior frontal gyrus (triangular part)

^a^ Significantly activated clusters with 10 or more voxels

^b^ p < 0.05 FWE-corrected at the cluster level

^c^ Coordinates in MNI space

^d^ Clusters were automatically labeled using the AAL toolbox [31].

3) Integrative network: The identified network included clusters in the orbitofrontal cortex, the insula, the inferior parietal lobule, and the middle and superior temporal gyrus (see Table 4). This network comprised several areas known to be involved in multisensory integration (hence the label), and showed a clear predominance for the left hemisphere, ipsilateral to the application of the stimuli. The comparison of stimulus-specific network patterns again showed no significant differences (see Fig. 1C).

**Table 4:** Significant FWE- corrected clusters of the chemosensory integration network for all substances separately and for the conjunction of three stimuli.

	Cluster size^a^	p- value^b^	x^c^	y^c^	z^c^	Anatomical label^c^
conjunction	3031	< 0.05	-42	22	-14	inferior frontal gyrus (orbital part)
	647	< 0.05	-60	-40	4	middle temporal gyrus
	49	< 0.05	-12	56	24	superior frontal gyrus
CO_2_	3663	< 0.05	-42	22	-14	inferior frontal gyrus (orbital part)
	1112	< 0.05	-50	-64	20	middle temporal gyrus
	152	< 0.05	-18	60	18	superior frontal gyrus
	32	< 0.05	38	14	-6	insula
	24	< 0.05	6	-64	60	precuneus
	15	< 0.05	50	16	0	middle temporal gyrus
	14	< 0.05	-42	-14	4	insula
	10	< 0.05	50	32	-2	inferior frontal gyrus (opercular part)
menthol	3628	< 0.05	-42	22	-14	inferior frontal gyrus (orbital part)
	967	< 0.05	-60	-40	4	middle temporal gyrus
	159	< 0.05	-12	56	24	superior frontal gyrus
	108	< 0.05	48	14	2	inferior frontal gyrus (opercular part)
	55	< 0.05	-38	0	46	precentral gyrus
	51	< 0.05	-62	-12	-16	middle temporal gyrus
	24	< 0.05	-62	-20	20	postcentral gyrus
	18	< 0.05	-10	8	42	cingular cortex
cinnamaldehyde	3703	< 0.05	-44	22	-14	inferior frontal gyrus (orbital part)
	1937	< 0.05	-60	-40	4	middle temporal gyrus
	222	< 0.05	-34	2	46	precentral gyrus
	95	< 0.05	-12	56	26	superior frontal gyrus
	38	< 0.05	-10	12	54	supplementary motor area
	37	< 0.05	-60	-16	20	postcentral gyrus
	23	< 0.05	50	16	0	inferior frontal gyrus (opercular part)

^a^ Significantly activated clusters with 10 or more voxels

^b^ p < 0.05 FWE-corrected at the cluster level

^c^ Coordinates in MNI space

^d^ Clusters were automatically labeled using the AAL toolbox [31].

## Discussion

In this study, we were able to show that distinct trigeminal chemoreceptors share the same processing pathway in the brain. Specifically, stimulation of three different TRP channels with relatively specific substances led to the activation of very similar brain networks. In all three stimuli, we observed three distinct networks, namely (1) an olfactory network, (2) a somatosensory network, and (3) an integrative network.

Results of previous studies have shown that TRPV1 and TRPA1 are often expressed in the same sensory neurons [16,17]. In contrast, TRPM8 is exclusively expressed in different sensory neurons [17]. Interestingly, Wang et al. [33] have shown that CO_2_, which is known to activate TRPV1, also evokes responses in TRPA1. These findings may suggest that the neuronal processing networks evoked by CO_2_ and cinnamaldehyde show a higher degree of concordance compared to the spatial network pattern induced by menthol. However, the results of our study revealed highly consistent networks for all three substances, indicating a common processing pathway of trigeminal stimuli.

In the following paragraphs, all three determined functional networks will be discussed in relation to our data and the published literature in detail.

### The olfactory network

The olfactory network includes clusters in areas related to odor processing, such as the PIR, the entorhinal cortex, the parahippocampal areas, and the insula. These findings are in line with previously published functional imaging data (for review see [34]). This network was determined for all investigated substances. It may be no surprise that menthol and cinnamaldehyde with their clear olfactory precepts evoked an olfactory network. It is, however, interesting to note that even CO_2_, which is a strong trigeminal stimulus (causing stinging or tingling sensations) with virtually no odor, activated this olfactory network—and to the same extent as the other two stimuli. The evaluation of the three stimuli revealed that the majority of raters were able to label menthol and cinnamaldehyde (cinnamon) correctly, whereas CO_2_ was labelled as ‘no specific odor’. This activation of olfactory areas evoked by CO_2_ result supports the close relationship of the olfactory and the trigeminal pathway, as suggested by previous studies [1,35–41]. A recently published study that used electrophysiological single- and multi-unit recordings may shed light on the impact of olfactory brain areas on trigeminal stimulus processing [42]. The neuronal activation pattern in the PIR of mice, evoked by CO_2_ stimulation, differed from the activation pattern induced by olfactory stimuli. This finding suggests that the PIR is responsible for the encoding of the stimulus modality (olfactory or trigeminal). In addition, CO_2_ stimulation led to a delay in PIR activation, indicating that trigeminal stimuli enter the PIR via a different route compared to olfactory stimuli. Unfortunately, fMRI is not appropriate for the examination of these temporal differences; however, it would be interesting to work on this question in future investigations. Thus, the PIR may be seen as a chemosensory processing area rather than a pure olfactory area.

### The somatosensory network

The second network we observed, covered parts of the so called “pain matrix” [32], a network which is involved in processing a large variety of painful sensations [43–45]. The core regions of this network are the primary and secondary somatosensory areas, the insula, and the anterior cingulate cortex [46–48]. In our study we were able to determine a network involving the primary and secondary somatosensory areas as well as the insula. Even though two of the applied stimuli were not perceived as painful, core regions of the pain network were activated in all three substances. The trigeminal pathway processes a variety of sensory inputs, such as temperature, somatosensory or nociceptive stimuli [49]. Hence, any sensory input which is processed via the trigeminal pathway is potentially harmful and painful and may therefore prepare and activate parts of the pain matrix.

In accordance with previously published findings investigating central processing of pain, we detected larger activation clusters in the right hemisphere contralateral to stimulation [50]. One possible reason for increased activation in the right hemisphere has been described in the homeostatic model of awareness, which claims an asymmetry of emotional awareness [51,52]. Here, both, positive emotions and sensory input are processed in the left anterior insula (AI), whereas negative emotions and sensations, such as pain, are processed predominantly in the right AI. However, our results argue against this interpretation, as we did not observe any significant differences in the neuronal activation pattern between the investigated stimuli; thus, even the non-painful stimuli (cinnamaldehyde and menthol) led to predominantly right-sided activations (see Fig. 1B).

### The integrative network

The detected network was lateralized to the left and included neuronal activation in the orbitofrontal gyrus, the inferior parietal lobule, the superior temporal gyrus, and a large cluster in the AI. These areas are involved in multisensory integration [52–54], particularly that involving chemosensory stimuli. In fact, the same brain areas were recently identified as constituting a task-independent network for the processing of mixed olfactory-trigeminal stimuli [19]. However, that study reported a predominance of the right hemisphere, in contrast to our findings. We would like to remind the reader that we stimulated participants exclusively through their left nostril, whereas the earlier study used bilateral stimulation. However, our observation of an ipsilateral network is in accordance with the notion that olfactory information is processed predominantly, but not exclusively, ipsilaterally [49].

## Conclusion

Within the last several years, the intranasal trigeminal system has been investigated in more detail, resulting in a deeper insight into the transduction of potentially noxious stimuli via the trigeminal pathway. Although previous studies identified different subfamilies of TRP channels, serving as receptors that evoke trigeminal sensations, the neuronal basis and processing pathway of these substances was still unclear. The results of our study suggest a common processing pathway for trigeminal stimulation, as the investigated substances targeted various sub-types of TRP channels and evoked distinct sensations in the nose. Thus, we assume that, although the TRPM8 receptor is exclusively found in different sensory neurons from the TRPV1 and TRPA1 receptors, the neuronal activation is processed in the same neuronal network.
